# Pathomechanisms in the Kidneys in Selected Protozoan Parasitic Infections

**DOI:** 10.3390/ijms22084209

**Published:** 2021-04-19

**Authors:** Karolina Kot, Natalia Łanocha-Arendarczyk, Michał Ptak, Aleksandra Łanocha, Elżbieta Kalisińska, Danuta Kosik-Bogacka

**Affiliations:** 1 Department of Biology and Medical Parasitology, Pomeranian Medical University in Szczecin, Powstańców Wielkopolskich 72, 70-111 Szczecin, Poland; kotkar@pum.edu.pl (K.K.); nlanocha@pum.edu.pl (N.Ł.-A.); ekalist@pum.edu.pl (E.K.); 2 Department of Nephrology, Transplantology and Internal Medicine, Pomeranian Medical University in Szczecin, Powstańców Wielkopolskich 72, 70-111 Szczecin, Poland; michalptak@mail.com; 3 Department of Haematology and Transplantology, Pomeranian Medical University in Szczecin, Unii Lubelskiej 1, 71-252 Szczecin, Poland; aleksandra.lanocha@pum.edu.pl; 4 Independent Laboratory of Pharmaceutical Botany, Pomeranian Medical University in Szczecin, Powstańców Wielkopolskich 72, 70-111 Szczecin, Poland

**Keywords:** *Acanthamoeba* spp., kidneys, *Leishmania* spp., *Plasmodium* spp., *Toxoplasma gondii*

## Abstract

Leishmaniasis, malaria, toxoplasmosis, and acanthamoebiasis are protozoan parasitic infections. They remain important contributors to the development of kidney disease, which is associated with increased patients’ morbidity and mortality. Kidney injury mechanisms are not fully understood in protozoan parasitic diseases, bringing major difficulties to specific therapeutic interventions. The aim of this review is to present the biochemical and molecular mechanisms in kidneys infected with *Leishmania* spp., *Plasmodium* spp., *Toxoplasma gondii*, and *Acanthamoeba* spp. We present available mechanisms of an immune response, oxidative stress, apoptosis process, hypoxia, biomarkers of renal injury in the serum or urine, and the histopathological changes of kidneys infected with the selected parasites. Pathomechanisms of *Leishmania* spp. and *Plasmodium* spp. infections have been deeply investigated, while *Toxoplasma gondii* and *Acanthamoeba* spp. infections in the kidneys are not well known yet. Deeper knowledge of kidney involvement in leishmaniasis and malaria by presenting their mechanisms provides insight into how to create novel and effective treatments. Additionally, the presented work shows gaps in the pathophysiology of renal toxoplasmosis and acanthamoebiasis, which need further research.

## 1. Introduction

Kidney diseases are increasingly recognized as a global public health problem. It is estimated that more than 850 million individuals have kidney disease, which is twice the calculated number of people with diabetes worldwide and >20 times higher than the number of individuals affected by AIDS/HIV globally [1].

One of the causes of kidney dysfunction is parasitic infection. Of the 342 parasites that infect humans, 20 are associated with kidney disease [2]. Parasites can cause nephropathy by different mechanisms: (i) by severe systemic infection; (ii) by activating the immune system, leading to glomerulonephritis, and (iii) by direct parasite infection in the urinary tract [2,3]. However, exact kidney injury mechanisms in parasitic infections are poorly known in many cases, bringing major difficulties to specific therapeutic interventions. Additionally, kidney involvement in parasitic diseases is almost always late, being an important cause of medical complications. The long-term impact of these infections on kidney tissue has never been investigated and might be a major cause of future kidney disease [4].

In the presented review, we focus on kidney involvement in protozoan parasitic infections. We decided to focus on the most important parasitosis in terms of clinical and epidemiological importance [5]. The paper presents the biochemical and molecular mechanisms in kidneys infected with *Leishmania* spp., *Plasmodium* spp., *Toxoplasma gondii*, and *Acanthamoeba* spp. based on the available data.

## 2. *Leishmania* spp.

Leishmaniasis is caused by an obligate intracellular parasite, which is endemic in subtropical and tropical countries. *Leishmania* spp. is transmitted through the bite of infected female sandflies, *Phlebotomus spp.* and *Lutzomyia spp.* [6,7]. *Leishmania* spp. are responsible for four different clinical forms of the disease: cutaneous leishmaniasis (CL), mucocutaneous leishmaniasis (ML), visceral leishmaniasis (also known as kala-azar, VL), and post-kala-azar dermal leishmaniasis [6,7].

Cutaneous and mucocutaneous leishmaniases are usually caused by *Leishmania tropica complex and L. braziliensis* complex, respectively. The diseases manifest with ulcers and nodules, which involve the skin as well as the respiratory and oral mucosa. Visceral leishmaniasis, usually caused by *Leishmania donovani* complex, is a systemic disease [8,9]. The onset of symptoms includes malaise, fatigue, fever, and weight loss. Due to *Leishmania* spp. tropism to the reticular endothelial system, hepatosplenomegaly, anemia, leukopenia with thrombocytopenia, and hypergammaglobulinemia are also seen in patients with VL. The disease can be fatal in almost 100% of the cases if left untreated [7,10]. Immunosuppressed patients may develop post-kala-azar dermal leishmaniasis, a complication of VL, that typically manifests with a macular or nodular rash that occurs after six months to one year after the systemic disease. VL may occur with varying clinical features, and the kidney can also be involved. Proximal and distal tubulopathy, acute glomerulonephritis, nephrotic syndrome, and acute kidney injury can develop in VL [11]. Both the disease itself and the therapy administered might be responsible for the renal involvement in VL. Some drugs used in visceral leishmaniasis, including amphotericin B, antimonial compounds, miltefosine, pentamidine, simataquine, and paromomycin, may be associated with a high risk of renal toxicity [6,8]. Kidney involvement in VL is associated with increased mortality, reaching ~95% [6].

### 2.1. Immune Response

A key role in the functioning of the immune system is played by innate response mechanisms, also known as the non-specific response. The activation and control of acquired immunity mechanisms also depend on innate responses. One of the most important issues in inducing an innate response is the ability of the immune system to distinguish “foreign” particles of a microorganism from “self” particles of the host organism. This task is performed by pattern recognition receptors (PRRs) found on monocytes and macrophages, neutrophils, dendritic cells, and epithelial cells. Examples of PRRs are Toll-like receptors (TLRs) [12,13]. The mechanism of TLRs is based on the recognition of pathogen-associated molecular patterns (PAMPs), which are compounds of exogenous origin, and danger-associated molecular patterns (DAMPs) that receive endogenous information [13]. Signal pathways triggered by TLRs lead to the activation of major transcription factors: nuclear factor қ-light chain-enhancer of activated B cells (NF-қB), activator protein-1 (AP-1), and interferon regulatory factors (IRFs) [14]. The first two of these factors trigger the expression of hundreds of genes responsible for the production of cytokines, chemokines, and pro-inflammatory enzymes. The most important compounds of this type include tumor necrosis factor α (TNF-α), interleukins (IL-1, IL-2, IL-6, IL-12), and chemokines (MIP-1 and MCP1, 2, and 3). However, the transcription factors IRFs regulate the expression of genes encoding type I interferons, IFN-α, and IFN-β [15].

Some studies have shown the role of TLRs in the pathogenesis of kidney diseases. It was found that interleukins and TNF-α, which are released after TLR activation, can cause glomerular damage [16]. Additionally, TLRs activate the adaptive immune system by antigen-presenting cells that promote CD4 helper cell differentiation [17]. CD4 Th1 and Th2 cells cause damage to the glomerular tissues mainly through basophils and macrophages, whereas Th17 cells may directly mediate damage to kidney structures in particular diseases [18]. Additionally, medical conditions involving the kidneys, such as acute kidney injury (AKI) or nephropathy, are related to inflammation that may be caused by DAMPs molecules [18,19]. These molecules may be released from dying renal cells or during modulation of the extracellular matrix [20,21].

The expression of TLR2 and TLR4 in the renal tissue of mice experimentally infected *Leishmania donovani* was studied by Kumar et al. [22] using real-time PCR. The TLR2 and TLR4 expressions were measured at 60- and 90-days post-infection (dpi). The mRNA levels of TLRs were similar at 60 and 90 dpi. At both, 60- and 90-days post-*L. donovani* infection, there was upregulation of TLR2 and TLR4. Kumar et al. [22] also analyzed the cytokine levels. On the 60th day post-*L. donovani* infection, the levels of TNF-α, IL-12, and IFN-γ were statistically higher as compared with the 90th day. The uninfected mice had lower levels of cytokine in the renal tissues compared with *L. donovani* infected hosts. The authors suggested activation of TLRs by *L. donovani* proteins in renal tissues that further augment TNF-α, IL-12, and IFN-γ production. The inflammatory cytokines propel initial injury and death of renal cells during *Leishmania* spp. infection [22]. Cytokines also influence leukocyte infiltration [23], and Costa et al. [24] characterized the inflammatory cells in the glomeruli of dogs naturally infected with *Leishmania* spp. CD4+ T cells were observed in the glomeruli of 80% dogs, while CD8+ T cells were noted only in 31% of dogs. In all cases exhibiting CD8+ T cells, CD4+ T cells were also found. No CD8+ T cells cytotoxicity in the *Leishmania* spp.-infected kidneys was found. However, a positive correlation was noted between *Leishmania* antigen and CD4+ T cells in the glomeruli. Costa et al. [24] suggested that a mixed Th1 and Th2 response is involved in extracellular parasite eradication. It seems that *Leishmania* spp. induce the production of growth factors, interleukins, and IFN-γ, which affect the cellular immune response and induce glomerular disease [24]. It is reported that immunoglobulins and complement do not take part in the development of nephropathy in VL because their levels were similar in infected and uninfected hosts infected with *Leishmania* spp. [24].

Macrophages, granulocytes, and natural killer cells (NK) are also involved in the innate immune response and participate in the genesis of glomerular lesions through a chain of cytokines and inflammatory mediators [25]. Zhou et al. [26] examined the influence of NK cells on the kidneys in leishmaniasis by analyzing SHP-1 phosphatase which blocks the cytotoxic reaction of NK cells and thus blocks innate immune responses [27]. Zhou et al. [26] examined SHP-1 activity in the kidney inner medulla of mice infected with *L. chagasi* using Western blot and immunohistochemistry methods. It was noted that *L. chagasi* increased SHP-1 activity in the inner medulla of kidneys. The authors also examined the phosphorylation of the tyrosines. Phosphorylation of Y536 usually activates the phosphate, whereas S591 phosphorylation often does the opposite. Zhou et al. [26] found that *L. chagasi* increased phosphorylation of SHP-1-Y536 in the renal cortex and inner medulla, while no effect was observed in SHP-1- S591. Based on the results of the study, Zhou et al. [26] reported that *L. chagasi* activates SHP-1 by phosphorylation of its Y536 region. Therefore, SHP-1 is involved in *Leishmania*-induced pathological effects in the kidneys.

### 2.2. Oxidative Stress

Under physiological conditions, reactive oxygen species (ROS), including superoxide anion (O_2_^**.**−^) and hydrogen peroxide (H_2_O_2_), are necessary for the proper functioning of cells and organisms in humans and animals because they are used by phagocytes to kill the bacteria the phagocytes have absorbed. Additionally, ROS play an important role in regulating cell signaling and genetic expression. The balance between the rate of free radical production and the level of antioxidants determines the level of ROS in the body and the speed of their reaction with cell components [28,29]. The disruption of homeostasis leading to an equilibrium shift toward ROS is called oxidative stress. This condition can occur as a result of over-generation of free radicals, or as a result of a decrease in the efficiency of defense systems, e.g., antioxidant levels. Mild oxygen shock is usually tolerated by the body, while its prolongation or intensification leads to numerous changes that are unfavorable to the cell, including disturbances in cell metabolism and switching of signals controlling apoptosis and necrosis. ROS can react with proteins, carbohydrates, lipids, and DNA [30]. Understanding the sources of their formation and the relationship with the level of antioxidant defense is one of the possibilities of explaining the molecular mechanisms of the development of many diseases, including kidney diseases [31].

Oxidative stress markers—O_2_^−^, H_2_O_2_, and malondialdehyde (MDA, the product of lipid peroxidation)—were measured in the kidneys of mice infected with *L. donovani* [22]. The levels of oxidative stress markers were statistically higher at 60 dpi compared with the control group and declined at 90 dpi. The results were correlated with histopathological observation of kidneys. Kumar et al. [22] reported that persistent production of ROS in VL may lead to organ or tissue damage by influencing the structure of proteins and by lipid peroxidation, which further alters the architecture of cells. It is important to point out that these radicals also help in the activation of the inflammatory response by regulating tyrosine and mitogen activated protein kinase (MAPK) and by activating transcriptional factors, which lead to the production of both inflammatory and anti-inflammatory cytokines.

### 2.3. Apoptosis

In the kidneys, apoptosis plays an important role not only during their development but also in the mature organ, enabling the replacement of unnecessary epithelial cells of the renal tubules. In a normal, mature kidney, apoptosis is relatively low; however, it can be greatly exacerbated in kidney damage or diseases. The presence of increased apoptosis in the renal tubules has been found in acute ischemic renal failure. Moreover, disturbances in the proper course of apoptosis may lead to the development of many kidney diseases, such as interstitial inflammation or renal fibrosis [32]. In parasitic diseases, apoptosis also plays an important role in pathogenesis [33].

Apoptosis is the process of programmed, suicidal cell death. It involves the internal pathway (mitochondrial, P53 dependent), the external pathway (receptor), the perforin and granzyme B pathways, and the endoplasmic reticulum pathway (stress-induced) [34,35]. Regardless of the pathway that is used, caspase group enzymes are activated in the cytoplasm of the dying cell. Due to the stage of the apoptotic process, in which individual enzymes are activated, they are divided into inducing caspases (activator; -2, -8, -9, -10, and -12) and executive caspases (effector; -3, -6, -7) [36]. A significant role in the process of apoptosis is played by caspase-3, the activation of which is necessary for all pathways by apoptotic signaling. The signal carried by caspases leads to the induction of transcription factors such as AP-1 and NF-κβ. As a consequence, many proteins that disrupt the structure and metabolic functions of the cell appear in the cytoplasm, which contributes to its death [37].

Apoptosis in the kidney of dogs naturally infected with *Leishmania* spp. was detected by Costa et al. [24] using two different methods: detection of the M30 cytodeath marker and the TUNEL method. Fewer apoptotic cells were found in the glomeruli of *Leishmania* spp.-infected dogs than in uninfected animals. The results obtained from the two methods were very similar. However, the authors reported that the results might be biased because, in the control samples, the frequency of cells undergoing apoptosis process was relatively high, which could be caused by contact of control animals with different pathogens present in the environment [24]. In 2017, Kumar et al. [22] analyzed in the kidneys of mice infected with *L. donovani* the activity of caspase-3 and the mRNA level of caspase-3 using caspase 3 fluorimetric assay kit and real-time PCR, respectively. The activity of caspase-3 was statistically higher in infected mice than in uninfected hosts, and it was statistically more elevated at 90 dpi than at 60 dpi. The levels of caspase-3 mRNA were measured at 60th and 90th day post *Leishmania* spp. infection. An upregulation of this enzyme at 60 and 90 dpi compared with uninfected animals was reported. Moreover, there was a statistically significant difference between 60 and 90 dpi—more than 2 times higher at 90 dpi. The study conducted by Kumar et al. [22] revealed that apoptosis plays a significant role in the development of the abnormal function of kidneys in *L. donovani* infection.

### 2.4. Transforming Growth Factor β (TGF-β)

Transforming growth factor -β (TGF-β) belongs to the cytokine group, which exert multifunctional effects on cell proliferation, migration, differentiation, extracellular matrix (ECM) production, and apoptosis [38]. TGF-β induces the tubular and glomerular epithelial cell to transition and induces excessive ECM production and deposition in glomeruli and tubulointerstitium. TGF-β is expressed in a wide range of kidney diseases associated with fibrosis. On the other hand, a number of studies have reported that TGF-β deficient mice suffered from lethal inflammation and early death, suggesting a protective role for TGF-β in renal inflammation. Thus, TGF-β may exert its diverse role in renal inflammation and fibrosis by interacting with many signaling pathways and molecules [39].

The TGF-β level in the kidneys of mice infected with *L. donovani* was assessed by Kumar et al. [22] using an ELISA assay kit. The TGF-β level in the kidneys of mice at 60 days post-*L. donovani* infection was slightly increased compared with the control group of mice. But at 90 dpi, the TGF-β level was significantly higher than at 60 dpi. Using the immunohistochemistry method, expression of TGF-β was also detected in the kidneys of dogs infected with *L. infantum* [40]. Higher expression was noted in the kidneys of dogs infected with the parasite compared with the control group. Histological sections of dogs’ kidneys stained with Masson’s trichrome confirmed fibroblast proliferation and collagen deposition in the tubules and the interstitium of the renal cortex and renal medulla layer. As the consequence of TGF-β increased expression and the tubular lesion, hypoxia may occur, which in turn can induce the apoptosis process [41,42]. For this reason, Kumar et al. [22] suggested that TGF-β may mediate apoptosis in the renal cells in the *Leishmania* spp.-infected hosts, which resulted in cellular disorganization and renal tissue damage.

### 2.5. Neutrophil Gelatinase-Associated Lipocalin (NGAL)

Neutrophil gelatinase-associated lipocalin-1, with a molecular weight of about 25 kDa, is a small protein belonging to the lipocalin family, originally detected in activated neutrophils as a gelatinase (type IV collagenase) protein. The NGAL-1 protein is synthesized in kidneys and other organs, including the prostate, trachea, lungs, stomach, large intestine, uterus, and bone marrow. Moreover, being an acute-phase protein, it is released in higher amounts from neutrophils and macrophages in inflammation and endothelial damage. Its source is also adipocytes [43].

NGAL undergoes glomerular filtration in the kidneys and reabsorption in the proximal tubules. The increased concentration of NGAL-1 in the urine may be a consequence of damage to the proximal tubules and a decrease in the renal clearance of this protein (reduced glomerular filtration). However, cells of the distal parts of nephrons, the Henle loop and distal tubules, can also activate NGAL-1 synthesis [44]. NGAL-1 is considered a good biomarker of kidney damage because the increased concentration of this protein in both plasma and urine is observed 2 h after the action of the kidney damaging factor. Moreover, it has been shown to predict adverse outcomes of chronic kidney disease, and it correlates with the degree of renal damage. NGAL has been linked with several conditions, including metabolic, immunological, and inflammatory diseases [45,46].

Using commercial ELISA kits, Peris et al. [47] measured NGAL in urine and serum samples (uNGAL and sNGAL, respectively) of dogs experimentally infected with *L. infantum*. The dogs were divided into four groups: non-proteinuric dogs infected with *L. infantum*, group 1; borderline proteinuric dogs infected with *L. infantum*, group 2; proteinuric dogs infected with *L. infantum*, group 3; and uninfected dogs, control group. No significant differences in sNGAL determination between the control group, group 1, group 2, and group 3 were reported. Additionally, there was no increase in sNGAL between groups 1 to 3 [47]. Taking into account urinary NGAL to creatinine ratio (uNGAL/C), there were statistically significant differences among groups: control vs. group 3 and group 1 vs. group 3. Moreover, uNGAL/C values increased from group 1 to group 3 [47]. The authors reported that uNGAL correlated with glomerular lesions. Meneses et al. [48] examined sNGAL and uNGAL in patients with VL using ELISA kits. The mean sNGAL level was statistically increased in the serum of patients with VL and acute kidney injury (AKI group) compared with patients with VL and without AKI (non-AKI group). Additionally, statistically significant differences were observed in the sNGAL level between the non-AKI group of patients compared with healthy participants. The mean uNGAL level was significantly higher only in the AKI group compared with healthy individuals. Meneses et al. [48] reported that sNGAL was associated with AKI development and clinical renal markers. Both Peris et al. [47] and Meneses et al. [48] suggested that NGAL can be considered as a good biomarker of leishmaniasis with kidney involvement.

### 2.6. Kidney Injury Molecule 1 (KIM-1)

The kidney injury molecule is a type 1 transmembrane glycoprotein with a molecular weight of approximately 104 kDa. It is located in the apical part of the proximal tubule of the nephron in the outer layer of the medulla. KIM-1 consists of an extracellular immunoglobulin domain, a middle mucin domain c, a single transmembrane domain, and a C-terminal cytoplasmic domain [49]. The extracellular domain of KIM-1 is cleaved by MMPs in response to tubular damage and inflammation, and the domain is released into the urine. KIM-1 is a quantitative indicator of kidney damage [50]. During normal renal function, KIM-1 is expressed at low levels in the glomeruli, periurethral interstitial cells, and internal medullary cells [51]. KIM-1 expression is significantly increased due to renal ischemia and damage to the renal tubules due to nephrotoxic factors [50]. In the early stage of kidney damage, KIM-1 has anti-inflammatory functions, mediating phagocytic processes in tubular cells [52]. There are also reports on the role of KIM-1 in the process of interstitial renal fibrosis. Renal tubules with a high content of KIM-1 are surrounded by inflammatory cells and have fibrotic features, and their glomerular and segmental sclerosis occurs [53,54]. KIM-1 is an early marker of kidney injury that shows increased expression before the onset of full-blown disease. In the urine of patients with toxic damage to the renal tubules, an at least 5-fold increase in KIM-1 can be observed on the first day after damage. Moreover, studies have shown that KIM-1 can serve as a marker of acute renal failure of various etiologies, including after cardiac surgery in extracorporeal circulation [55], in sepsis [56], and after kidney transplantation [57]. Increased levels of KIM-1 were also observed in the urine of patients with chronic renal failure [58] and with clear cell kidney cancer [59].

Meneses et al. [48] examined urine KIM-1 (uKIM-1) in patients with VL using ELISA kits. The mean uKIM-1 level was above 3 times higher in patients with VL and acute kidney injury (AKI group) compared with healthy individuals, above 2 times higher in patients with VL and without AKI (non-AKI group) compared with healthy individuals, and almost 1.5 times higher in AKI group compared with the non-AKI group. However, a statistically significant difference was noted only between the AKI group vs. healthy patients. Meneses et al. [48] suggest that patients infected with *Leishmania* spp. had an injury in renal proximal tubules and that KIM-1 is not a promising biomarker for early detection of nephropathy in VL.

### 2.7. Monocyte Chemoattractant Protein-1 (MCP-1)

Monocyte chemoattractant protein-1 (also known as chemokine (C-C motif) ligand 2, CCL-2) is known to be an important mediator of innate immunity and tissue inflammation [60]. MCP-1 is secreted in response to signals, such as IL-1, TNF-α, INF-γ, TGF-β, ROS, and it plays an important role in selectively recruiting monocytes, neutrophils, and lymphocytes. MCP-1 is a potent chemotactic factor for monocytes, and it plays an important role in various pathophysiological conditions in many organ systems. In the kidney, cell types producing MCP-1 are tubular cells, smooth muscle cells, mesangial cells, podocytes, and also infiltrating cells such as eosinophils and mast cells [61,62]. MCP-1 is used as a biomarker of histopathological changes in kidneys, of lupus nephropathy, and of the presence of tubulointerstitial changes and the advancement of chronic changes in kidneys [63]. There are little data in the literature reporting on the association between parasitic diseases and MCP-1.

Oliveira et al. [64] examined urine MCP-1 (uMCP-1) in patients with VL using ELISA kits. The uMCP-1 was significantly elevated in the patients with VL compared with the control group of patients. Meneses et al. [48] also examined uMCP-1 in patients with VL using ELISA kits; however, the patients with VL were grouped into two: with AKI (AKI group) and without AKI (non-AKI group). The mean uMCP-1 level was above 5.5 times higher in the AKI group compared with healthy individuals, above 3 times higher in the non-AKI group compared with the control group, and ~1.75 times higher in the AKI group compared with the non-AKI group. However, a statistically significant difference was noted only between the AKI group vs. healthy patients. Even though uMCP-1 involvement in renal inflammation and nephropathy in patients with VL was reported by Oliveira et al. [64], Meneses et al. [48] reported that uMCP-1 was not related to AKI development in patients infected with *Leishmania* spp.

### 2.8. Biochemical Parameters in the Serum and Urine

Biochemical parameters in the serum and urine, which allow evaluation of the condition and function of the kidneys, are called a renal panel. The examinations that are involved in the renal panel are a urine test, serum sodium (Na), serum potassium (K), serum urea, serum creatinine (CREA), serum uric acid (UA), and total serum protein (TP) [65]. Patients with kidney involvement in VL have hematuria, mild to modern proteinuria, and increased urine leukocytes in over 50% of cases. Creatine level is elevated or at normal level in the serum of patients with VL and renal involvement [66,67]. Peris et al. [47] examined urea, CREA, and TP in the serum of dogs experimentally infected with *L. infantum*. The authors reported a 2.5 times higher level of urea in dogs at 360 dpi and increased CREA in dogs at 300 and 360 dpi. However, no azotemia (creatine > 1.4 mg/dl) was found in any dogs [47]. Kumar et al. [22] reported an increased level of protein in the urine and increased CREA in the mice infected with *L. donovani* compared with control animals. However, the authors noted a statistically significant decrease in the level of protein in the urine and serum creatinine in the 90-day post-*L. donovani* infection compared with 60 dpi [22]. The divergent results of examinations make the interpretation of changes in the renal panel difficult.

### 2.9. Histopathological Changes

Peris et al. [47] observed glomerulonephritis in the kidneys of dogs experimentally infected with *L. infantum*, including membranoproliferative glomerulonephritis (MPGN) and mesangioproliferative glomerulonephritis (PGN). The authors reported that 54% of dogs had less than 50% of glomeruli injured, while 46% had more than 50% of glomeruli injured [47]. Kumar et al. [22] observed increased hypercellularity and reduced Bowman’s space at 60 dpi in mice experimentally infected with *L. donovani*. At 90 dpi, the glomerular cellularity was reduced.

**Figure 1:**
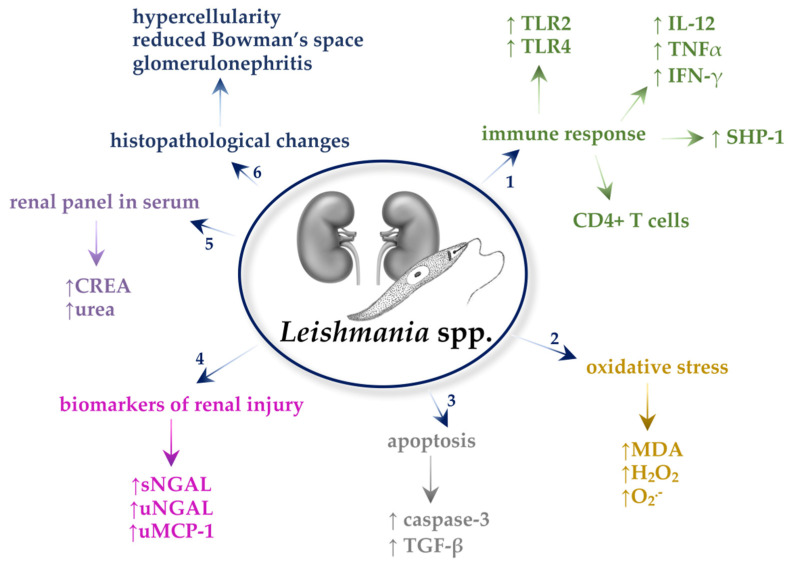
Mechanisms in *Leishmania* spp.-infected kidneys. 1. *Leishmania* spp. increase the expression of Toll-like receptors (TLRs), mainly TLR2 and TLR4, and they also affect the growth of interleukin-12 (IL-12), tumor necrosis factor-α (TNF-α), and interferon-γ (IFN-γ). It was found that *Leishmania* spp. increase SHP-1 activity in the kidneys of hosts. The inflammatory cells, which are observed in the kidneys of hosts infected with *Leishmania* spp., are CD4+ T cells. 2. *Leishmania* spp. cause oxidative stress manifested by an increase in malondialdehyde (MDA), H_2_O_2_, and O_2_^–^. 3. *Leishmania* spp. cause an increase in caspase-3 activity, the main enzyme in the process of apoptosis, and an increase in transforming growth factor β (TGF-β) level, an apoptotic inducer. 4. Biomarkers, which increase in nephropathy caused by *Leishmania* spp., are serum and urine neutrophil gelatinase-associated lipocalin (sNGAL and uNGAL, respectively) and monocyte chemoattractant protein-1 in the urine (uMCP-1). 5. *Leishmania* spp. cause an increase in creatine (CREA) and urea levels in the serum of hosts. 6. The parasites cause hypercellularity, reduced Bowman’s space, and glomerulonephritis in the kidneys.

All described mechanisms in *Leishmania* spp.-infected kidneys are presented in Figure 1.

## 3. *Plasmodium* spp.

Malaria is an infectious disease caused by the intracellular protozoa of the genus *Plasmodium*. The parasite has two hosts in its life cycle: the female mosquito of the genus *Anopheles*, which is the definitive host and at the same time the vector, and the human, who is the intermediate host for the parasite. Malaria in humans is caused by six *Plasmodium* species: *Plasmodium vivax*, *P. ovale curtisi*, *P. ovale wallikeri*, *P. malariae*, *P. falciparum*, and *P. knowlesi* [68].

*Plasmodium* spp. infection may be acute, fulminant, or chronic, involving multiple internal organs and systems. The symptoms of malaria may vary depending on the species of *Plasmodium* but generally are associated with headache, nausea, vomiting, chills and sweating, anemia, and hepatosplenomegaly [69]. Some authors suggest that even 60% of patients with severe malaria develop AKI [70]. Sacomboio et al. [71] observed that the parasitemia level in patients with malaria was increased in patients with mild and severe AKI. Moreover, the mortality rate was higher in patients with more advanced stages of kidney injury [71]. It is estimated that the mortality rate is 15–45% in patients with malaria and AKI. Early detection of malaria- kidney injury may help to avoid nephrotoxic drugs, prevent deterioration of kidney function by appropriate fluid management, and monitor the need for renal replacement therapy [72].

In scientific literature, three hypotheses exist about mechanisms of AKI in *Plasmodium* spp. infection: (i) Mechanical perturbations, which point that kidney injury is caused by intravascular clusters, which disturb blood flow to the kidneys leading to hypoxia. The clusters are created by parasitized red blood cells that adhere to healthy erythrocytes and thrombocytes [73]; (ii) Immune-mediated glomerular injury, which suggests that the parasite’s red blood cells activate blood mononuclear cells, which in turn causes the release of various cytokines. Interleukins and activated T cells drive activation of complement with immune complex deposition, which induces interstitial nephritis and glomerulonephritis [74]; (iii) Metabolic disturbances, which suggest that the parasite changes the host red blood cell membrane by a change in erythrocyte magnesium-activated ATPase. The change in red blood cell structures, by a decrease in Na concentration and increase in Ca level in the cell, results in organ sequestration that leads to kidney injury [75].

### 3.1. Immune Responses

One of PRR that plays a critical role in the host immune responses is lectin-like oxidized low-density lipoprotein receptor 1 (LOX-1). LOX-1 is a transmembrane protein presented in endothelial cells, macrophages, and monocytes. The receptor can facilitate the uptake of dying cells and cross-presentation of cellular antigen via binding with heat shock protein [76]. Under physiological conditions, LOX-1 expression is minimal but can be induced in various disorders, including damaged kidneys [77,78]. The stimuli include TNF-α, oxidized LDL, and oxidative stress [79,80]. Using real-time PCR, Elias et al. [81] detected LOX-1 mRNA expression in the renal tissue of mice infected with *Plasmodium* spp. The expression of LOX-1 was not detected until 6 days post-*Plasmodium* spp. infection. The statistically significant differences were between control mice and the *Plasmodium* spp.-infected mice at 6, 7, and 12 dpi. Probably, *Plasmodium* spp. infection generates ROS [82], which stimulate LOX-1 to gene expression.

The cytokines panel, including IL-1β, IL-6, IL-10, IFN-γ, and TNF-α, in renal tissue of mice infected with *Plasmodium* spp. was examined by Elias et al. [81] using a cytokine assay kit. IL-1β was statistically different only between 6 dpi and the uninfected mice. IL-6 level in the renal tissue was observed only at 6- and 7-days post-*Plasmodium* spp. infection. For the rest of the days, this cytokine was not detected. No significant differences in IL-6 level were reported. Renal protein expression of IL-10 from mice infected with *Plasmodium* spp. was decreasing during the infection. Statistically lower IL-10 expression was noted at 6 and 7 dpi compared with the control group of mice [81]. IFN-γ mRNA expression was not detected until 5 dpi. The statistically higher expression compared with control mice was found at 5 and 7 dpi. TNF-α increased, reaching a maximum on day 6 and then levels of this cytokine began to decline. A statistically significant difference was observed only at 6 dpi vs. the control group. The cytokine panel was also examined by Sinniah et al. [83] using reverse transcription PCR and immunohistochemistry methods. Pro-inflammatory cytokines IL-6 and TNF-α were also upregulated both at mRNA and at protein levels during the course of malaria nephropathy. The IFN-γ level increased only at the early stages of infection and then decreased to normal level after 10 dpi, but the mRNA levels were elevated in the kidneys during the *Plasmodium* spp. infection. Sinniah et al. [83] also examined immunoglobulin (Ig)A, IgM, and IgG immunohistochemistry staining in the kidneys of *Plasmodium* spp.-infected mice. The deposition of all studied immunoglobulins was reported in the glomeruli. Based on the study by Elias et al. [81] and Sinniah et al. [83], there are three hypotheses of immune mechanisms in kidneys: (i) lipopolysaccharide-like malaria antigen may induce the production of proinflammatory cytokines by macrophages in the kidneys and induce the inhibition of anti-inflammatory cytokines, IL-10; (ii) the higher secretion of proinflammatory cytokines is regulated by a higher level of IFN-γ; or (iii) the production of cytokines may be induced by the immune complex, which is deposited in the glomeruli of mice infected with *Plasmodium* spp.

### 3.2. Matrix Metalloproteinases (MMPs)

Matrix metalloproteinases (MMPs) are a large group of zinc ion-dependent enzymes. Currently, more than 20 metalloproteinases have been identified. Metalloproteinases are produced in leukocytes, macrophages, vascular endothelial cells, and most connective tissue cells. They are synthesized in an inactive form, and their activation occurs by breaking the bond between the cysteine residue and the zinc atom within the enzyme (reversible activation) or through limited proteolysis (irreversible activation) [84]. The expression and activation of MMPs are controlled mainly by the action of tissue inhibitors of metalloproteinases (TIMPs), which bind them non-covalently. To date, four types of TIMPs (TIMP1-4) have been identified [85,86]. The inhibition of MMPs activity is also caused by non-specific inhibitors, including α2-macroglobulin, C-terminal fragment of procollagen proteinase inhibiting MMP-2, corticosteroids, and interleukin 4 (IL-4) [87,88]. MMPs activation is also controlled by regulation at the level of gene transcription [89].

Metalloproteinases are proteins that play many important roles in biological processes. Their proteolytic activity significantly affects the composition and structure of the extracellular matrix (ECM) through the degradation of its components and the regulation of signaling particles. MMPs participate in the regulation of cells for differentiation, proliferation, apoptosis, adhesion, and migration, ensuring normal renal glomerular and tubular function [88,90,91]. In the kidney, MMPs are expressed in the glomerulus, proximal and distal tubules, and collecting tubules [88,90]. MMPs are involved in both physiological and pathological processes of the kidneys. MMP-2 and MMP-9 are synthesized in the first step of renal embryogenesis in vivo [92]. Moreover, MMP-9 protects mesenchymal cells against apoptosis and stimulates morphogenesis during kidney development by releasing the stem cell factor [93,94]. MMPs are involved in pathological processes such as AKI, chronic kidney disease, diabetic nephropathy, lupus nephritis, post-infectious glomerulonephritis, and chronic transplant rejection [91,95,96]. New data indicate that some MMPs also play a significant role in the pathogenesis of kidney fibrosis [97].

MMPs and TIMPs in the kidneys of mice infected with *Plasmodium* spp. were analyzed by Van den Steen et al. [98]. The hosts were injected intraperitoneally with *P. berghei*, and biochemical analyses were performed using quantitative RT-PCR and zymography. In the kidneys of mice, the authors reported decreased MMP-2 and MMP-11 at 6 and 8 days of disease development and decreased level of MMP-15 at 8 dpi. Additionally, the authors observed an increased level of MMP-14 at 6 and 8 dpi and also increased MMP-9 in the kidneys while the disease progressed (8 dpi). No changes in the expression of TIMPs in the kidneys between infected and uninfected animals were noted [98]. Punsawad and Viriyavejakul [99] analyzed the expression of MMP-3 in the kidneys of fatal *Plasmodium falciparum* cases using immunohistochemical staining. Patients were divided into 3 groups: (i) AKI group of patients infected with *P. falciparum*, (ii) non-AKI group of patients infected with *P. falciparum*, and (iii) control group. Immunoexpression of MMP-3 was detected only in the cytoplasm of the proximal tubular cells presenting with necrosis. The MMP-3 level was significantly higher in the kidney tissues of patients from the AKI group compared with non-AKI and control groups. Punsawad and Viriyavejakul [99] reported that mechanisms of MMP-3 activation in the kidneys in malaria are still unclear. It might be activated by mitogen-activated protein kinase (MAPK), by ROS, or by pro-inflammatory cytokines, particularly TNF-α [100,101]. Based on the studies conducted by Elias et al. [81] and Sinniah et al. [83], it is suggested that TNF-α is involved in tubular injury and might activate MMP-3 expression in malarial acute kidney injury.

The exact role of the different MMPs and TIMPs in the kidney in malaria infection has not yet been clarified. It is suggested that MMPs contribute to antimicrobial responses and that they may help in the apoptosis process [98].

### 3.3. Oxidative Stress

*Plasmodium* spp. generate abnormally large quantities of ROS in the infected red blood cells. Additionally, ROS is also generated as a result of the host’s immune response, and they have a protective effect against *Plasmodium* spp., but they also bring extensive damage to organs, something observed often in severe malaria [82].

Sharma et al. [102] used various methods to determine oxidant and antioxidant levels in the kidneys of mice infected with *Plasmodium* spp. The authors reported a statistically significant increased level of MDA in the kidneys of mice infected with *Plasmodium* spp. compared with uninfected animals. Additionally, the authors reported a statistically significant increased level of reduced glutathione (GSH) as well as superoxide dismutase (SOD) and a decreased level of catalase (CAT) in the kidneys of infected animals compared with the control group. In humans, Nanda et al. [103] examined MDA level in the serum of patients infected with *Plasmodium* spp. and with and without acute renal failure. The MDA level was statistically significantly higher in patients infected with *Plasmodium* spp. with acute renal failure compared with uncomplicated malaria cases. A positive correlation between serum MDA level and serum urea as well as CREA was reported. The product of lipid peroxidation can be responsible for mitochondrial disintegration, combined with depressed energy metabolism and diminished transport performance of the nephron. The authors suggested that MDA level in the serum of patients with malaria can be attributed to MDA level being a measure of severity of tissue damage [103].

The changes in antioxidant levels may be a consequence of oxidative stress, which is manifested by an increase in MDA levels. Moreover, if the antioxidative system is insufficient, cellular dysfunction and apoptosis can occur. Therefore, oxidative stress may be one of the mechanisms of nephropathy in malaria infection.

### 3.4. Apoptosis

Wichapoon et al. [104] reported the expression of cleaved caspase-3 in renal tubular cells in *Plasmodium falciparum* patients using the immunohistochemistry method. Apoptosis was the highest in the distal convoluted tubules. The percentage of apoptosis in the tubular cells was statistically significantly higher in the AKI group compared with the non-AKI and control group. Apoptosis rate in the non-AKI group was slightly higher than in the control group, but the difference was not statistically significant. The authors reported that cleaved caspase-3 immunoexpression was correlated with renal dysfunction, disease severity, as well as acute tubular necrosis. Apoptosis, performed by immunohistochemistry with cleaved caspase-3 antibody, was also detected in the kidneys of mice infected with *Plasmodium* spp. when compared with uninfected animals [81].

### 3.5. Hypoxia

Hypoxia denotes an environment characterized by oxygen deficiency. Hypoxia occurs when there is impaired blood transport to cells and organs. In kidneys, hypoxia plays a role in the pathogenesis of renal diseases. The kidneys show a remarkable discrepancy between blood supply and oxygenation. Despite high blood flow and oxygen delivery, oxygen tensions in the kidney are comparatively low. The constraint of oxygen supply to renal tissue makes the kidneys sensitive to hypoxia and has long been known as a significant factor in the pathogenesis of kidney diseases [105]. Hypoxia-inducible factor 1 (HIF-1), which controls the expression of numerous genes, is mainly responsible for the body’s response to hypoxia. The main task of HIF-1 is to switch aerobic metabolism to anaerobic energy production [106]. HIF-1 mRNA expression in the renal tissue of mice infected with *Plasmodium* spp. was performed by Elias et al. [81] using real-time PCR. Increased HIF-1 expression was observed in the renal tissue of mice at 3-, 4-, 6-, and 12-days post *Plasmodium* spp. infection compared with the uninfected animals. The upregulation of HIF-1 mRNA expression in the renal tissue of hosts with malaria can induce a morphological modification in the kidneys. Changes in vascular permeability in malaria-associated AKI are expected since microvascular dysfunction has been described in malaria [107].

### 3.6. Neutrophil Gelatinase-Associated Lipocalin (NGAL)

In a pilot study, van Wolfswinkel et al. [108] examined sNGAL and uNGAL in patients with imported *P. falciparum* using commercial ELISA kits. Six patients developed AKI. sNGAL was elevated in five patients with AKI, while uNGAL was elevated in all participants with malaria-induced AKI. sNGAL and uNGAL were significantly higher in the AKI group compared with the non-AKI group of patients. Thus, van Wolfswinkel et al. [108] suggested that sNGAL and uNGAL have an excellent predictive performance for malaria-induced AKI. uNGAL was also examined in patients with severe *Plasmodium falciparum* infection [109]. Plewes et al. [109] examined urine NGAL-corrected urine creatinine (uNGAL/Ucr) using ELISA kits, and they observed statistically significantly higher levels of uNGAL/Ucr in the patients with severe AKI compared with patients with moderate AKI, mild AKI, and without AKI. The authors suggested that NGAL is a good biomarker of AKI in severe falciparum malaria because its excretion is continued with ongoing renal stress.

### 3.7. Kidney Injury Molecule-1 (KIM-1)

Punsawad and Viriyavejakul [99] determined KIM-1 in the kidney tissues from fatal *P. falciparum* cases with and without AKI using immunohistochemical staining. KIM-1 was localized in the cytoplasm of the proximal tubular cells, but it was not detected in the glomeruli. The authors reported higher immunoexpression of KIM-1 in the AKI group compared with the non-AKI and control group of patients. Moreover, a positive relationship between KIM-1 expression and the percentage of proximal tubular necrosis was noted. Punsawad and Viriyavejakul [99] demonstrated that KIM-1 correlated with the degree of renal dysfunction and that it can be involved in the pathogenesis of proximal tubular cell damage and repair in AKI with severe *P. falciparum* infection. While van Wolfswinkel et al. [108] examined KIM-1 in the urine (uKIM-1) of patients with imported *P. falciparum* using a commercial ELISA kit. Fifteen percent of the patients developed AKI. Only one patient showed elevated uKIM-1 levels. The patient required renal replacement therapy (RRT). The authors, similar to Punsawad and Viriyavejakul [99], also reported that KIM-1 is a promising biomarker of malaria-induced AKI; however, larger studies are needed [108].

### 3.8. Monocyte Chemoattractant Protein-1 (MCP-1)

Renal tissue protein expression of MCP-1 during *Plasmodium* spp. infection was detected by Elias et al. [81] using a cytokine assay kit. Statistically significant elevated MCP-1 levels were observed at 6- and 7-days post-infection compared with the control group of mice. The upregulation of MCP-1 in the kidneys of *Plasmodium* spp.-infected mice suggested that mostly neutrophils, macrophages, and polymorphonuclear leucocytes infiltrate kidneys in *Plasmodium* spp. infection. Additionally, it is proposed that MCP-1 might be a good biomarker of nephropathy in malaria [81].

### 3.9. Renal Panel in the Blood

Examining the plasma of mice infected with *Plasmodium* spp., Elias et al. [81] assessed creatinine level and blood urea nitrogen (BUN) using Jaffe’s modified method and a Labtest Kit, respectively. Creatine level was increasing every day post-*Plasmodium* spp. infection, but statistically significant differences were observed at 7 and 12 dpi compared with the control group. BUN levels increased, then decreased, and again increased during the infection. The statistically significant differences were observed at 4, 5, 6, 7, and 12 dpi compared with the control group of mice. The authors found a correlation between the parasitemia level and the creatine level in the *Plasmodium* spp.-infected mice [81].

### 3.10. Histopathological Changes

In the kidneys of mice infected with *Plasmodium* spp., Elias et al. [81] observed changes in renal architecture that ranged from a mild mononuclear cell infiltration at 7 dpi to outstanding proinflammatory hypercellularity at 15 dpi. The morphological analysis of the kidney section showed changes that characterized acute tubule-interstitial nephritis [81]. Sinniah et al. [83] also reported morphological abnormalities during the early stages of infection in the kidneys of mice infected with *Plasmodium* spp. From day 10 dpi, an increased number of parasitized erythrocytes, lymphocytes, monocytes, macrophages, and polymorphonuclear leukocytes were seen in the glomeruli. Wichapoon et al. [110] examined histopathological changes of kidneys in fatal *P. falciparum* cases. In the *P. falciparum*-infected patients who also had AKI, thickening of basement membrane congestion, protein deposition in the Bowman’s capsule, the presence of parasitized red blood cells within the capillaries, and interstitial inflammation were observed. Additionally, swelling of tubular cells, tubular degeneration, and tubular necrosis were also reported. In the kidneys of patients with AKI, an increased number of glomerular cells and decreased glomerular area were noted compared with control kidneys and patients infected with *P. falciparum* without AKI. The authors explained the results by damaged tight junction-associated protein zonula occludens-1 (ZO-1) in the kidneys of *P. falciparum*-infected patients with AKI. The loss of tight junction-associated protein was demonstrated by the decrease in ZO-1 expression in the immunohistochemical examination, and the damaged cell junction was presented by immunofluorescence examination [110]. ZO-1 plays an important role in stabilizing the tight junction complex structure and signaling transduction [111]. In a different study, Wichapoon et al. [104] observed chronic inflammatory cells infiltrations, scattered with the diffused detachment of the renal tubular cells from the basement membrane, and deposition of hemosiderin pigments and the presence of packed red blood cells (PRBCs) in the kidneys of patients infected with *P. falciparum*. Tubular necrosis, which was confirmed based on the loss of tubular nuclei, detachment of basement membrane, tubulorrhexis, and dissociation of brush border, was quantified in the proximal and distal convoluted tubules (PCT and DCT, respectively) and collecting ducts (CD) of kidneys from patients with *P. falciparum* infection and AKI, patients with *P. falciparum* infection and without AKI, and patients without *P. falciparum* infection and without AKI. The highest renal tubular necrosis in all tubular segments was seen in the AKI group, affecting mostly the DCT. Renal tubular necrosis was increased in PCT and DCT in the AKI group compared with non-AKI and control groups. In the CD, statistically significant differences in the percentage of necrosis were observed between infected patients with AKI and the control group, and between infected patients without AKI and the control group [104].

**Figure 2:**
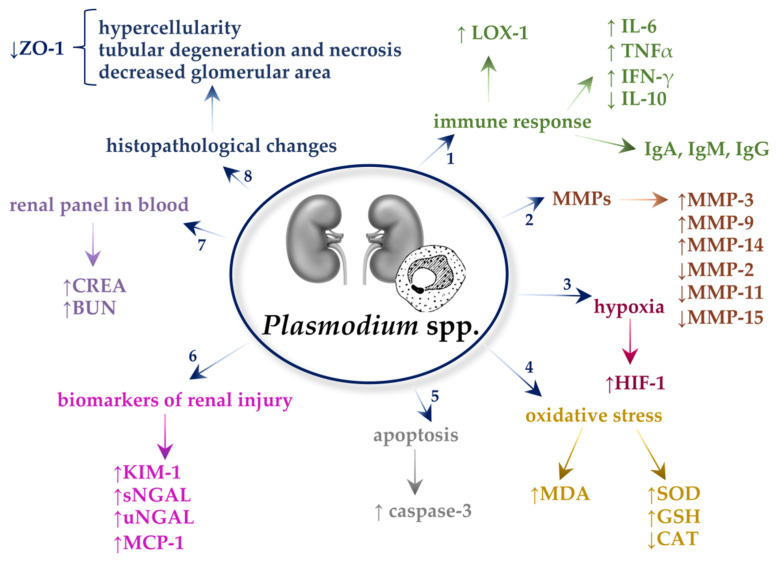
Mechanisms in *Plasmodium* spp.-infected kidneys. 1. *Plasmodium* spp. increase the expression of lectin-like oxidized low-density lipoprotein receptor 1 (LOX-1), and they also affect the growth of interleukin-6 (IL-6), tumor necrosis factor-α (TNF-α), and interferon- γ (IFN-γ). It was found that *Plasmodium* spp. decrease the level of the anti-inflammatory cytokine IL-10. The deposition of immunoglobulin (Ig)A, IgM, and IgG was reported in the glomeruli of mice infected with *Plasmodium* spp. 2. Malaria causes an increase in matrix metalloproteinase (MMP)-3, MMP-9, and MMP-14 and a decrease in MMP-2, MMP-11, and MMP-15. 3. The *Plasmodium* spp. infection causes hypoxia in the kidneys by increasing the hypoxia-inducible factor 1 (HIP-1). 4. *Plasmodium* spp. cause oxidative stress manifested by an increase in malondialdehyde (MDA) and an increase in superoxide dismutase (SOD), as well as reduced glutathione (GSH) and a decrease in catalase (CAT). 5. *Plasmodium* spp. cause an increase in caspase-3 activity, the main enzyme in the process of apoptosis. 6. Biomarkers, which increase in nephropathy caused by *Plasmodium* spp., are serum and urine neutrophil gelatinase-associated lipocalin (sNGAL and uNGAL, respectively) and monocyte chemoattractant protein-1 (MCP-1) and kidney injury molecule 1 (KIM-1) in kidneys. 7. *Plasmodium* spp. cause an increase in creatine (CREA) and blood urea nitrogen (BUN) levels in the blood of hosts. 6. The parasites cause hypercellularity, tubular degeneration, and necrosis and also decreased glomerular area, which may be caused by decreased tight junction-associated protein, zonula occludens-1 (ZO-1) in the kidneys.

All described mechanisms in *Plasmodium* spp.-infected kidneys are presented in Figure 2.

## 4. *Toxoplasma gondii*

Toxoplasmosis is a zoonotic parasitic infection caused by *Toxoplasma gondii*, an obligate, intracellular, apicomplexan protozoa. Toxoplasmosis is a parasitic disease widely distributed throughout the world [112,113]. The parasite has two hosts in its life cycle. The definitive hosts are members of family Falidae, while the intermediate hosts are mostly many mammals and birds [114]. *T. gondii* is transmitted by ingestion of water, fruits, and vegetables contaminated with sporulated oocysts; through consumption of tissue cysts in undercooked meat of an infected animal; congenitally via the placenta; through blood transfusion; and from organ transplant [112,114].

Toxoplasmosis in immunocompetent individuals is mostly asymptomatic, or with non-specific flu-like symptoms, but a burgeoning number of epidemiological studies suggest that the parasite can be associated with several long-term behavioral effects [115]. The infection is considered to be an opportunistic and life-threatening disease in immunocompromised people, pregnant women, and newborns. Primary *T. gondii* infection during pregnancy can result in congenital toxoplasmosis with abortion, neonatal death, chorioretinitis, and neurological disorders in neonates [116]. In immunosuppressed patients, the involvement of many organs and systems can occur following the infection. Most cases of toxoplasmosis in immunosuppressed patients result from secondary reactivation of latent infection because parasites are formed as a tissue cyst in many organs. It has been found that *T. gondii* has a tropism to the kidneys. The large number of parasites in the kidneys is probably associated with renal function and glomerular filtration [117]. The pathologic links between *T. gondii* infection and renal diseases have not yet been established. However, toxoplasmosis secondary to renal transplantation is associated with significant morbidity. The median time to diagnosis of toxoplasmosis following renal transplantation is about 92 days, and the mortality rate is between 14% and 50% [118,119].

### 4.1. Immune Responses

TLRs, in particular TLR2, have a protective role in kidney function against *T. gondii* infection. Kudo et al. [120] analyzed renal function (using GLDH methods with an N-assay BUN-L kit and a CRE-S kit) and the histopathological changes in the kidneys of TLR2-deficient and TLR4-deficient mice. Serum CREA and blood urea nitrogen were significantly elevated in both *T. gondii*-infected TLR-2-deficient and *T. gondii*-infected TLR-4-deficient mice compared with the control. In histological examination, TLR-2-deficient mice revealed significant renal damage by *T. gondii* infection. In the kidneys of TLR-2-deficient mice infected with the parasite, the authors observed glomerular tissue proliferation, extracellular matrix and glomerular swelling, vacuolization of tubules, and thickened Bowman’s capsules. In the kidneys of TLR-4-deficient mice infected with *T. gondii*, Kudo et al. [120] observed similar changes but the involvement was less severe.

Gamma interferon (IFN-γ) is a cytokine critical to both innate and adaptive immunity. The cytokine coordinates a diverse array of cellular programs through transcriptional regulation of the immunologically relevant gene. IFN-γ is secreted by activated T cells, natural killer cells, and dendritic cells [121]. It has been shown that IFN-γ inhibits the replication of *T. gondii* in the kidneys [122]. Norose et al. [122] found the levels of *T. gondii* loads in the kidneys of IFN-γ knockout mice were much higher than those in the kidneys of wild-type mice. Kudo et al. [120] also examined the role of IFN-γ in the kidneys of IFN-γ knockout mice infected with *T. gondii*. The authors examined pathological changes and renal function of *T. gondii*-infected IFN-γ knockout mice. Serum creatine, blood urine nitrogen (BUN), urine albumin/creatine ratio, and urine alpha-1-microglobulin did not reveal any significant changes in the levels between IFN-γ knockout mice and wild-type mice. Moreover, the histopathological changes were not significantly different between the two studied groups of mice. Kudo et al. [120], contrary to Norose et al. [122], indicated that IFN-γ does not play a role in the protection of the renal function against *T. gondii* infection.

### 4.2. Oxidative Stress

Antioxidant levels in the kidneys of rats infected with *T. gondii* were determined by Türkoglu et al. [123]. The authors measured CAT, glutathione peroxidase (GSH-Px), and SOD levels using commercially available ELISA kits after 30 days post-*T. gondii* infection. The SOD and GSH-Px in the kidneys were at a similar level between *T. gondii*-infected rats and the control group of animals, whereas CAT levels were higher in the kidneys of infected than in uninfected rats, but the difference was not statistically significant [123]. Based on the study, it is suggested that pro- and antioxidant potential is not disturbed in renal toxoplasmosis.

### 4.3. Apoptosis

Apoptosis in kidneys infected with *T. gondii* was studied only by Gharadaghi et al. [124]. They used the TUNEL method to examine apoptosis in the kidneys of rats infected with *T. gondii*. The percentage of apoptotic cells was above 2 times higher in the kidneys of *T. gondii* infected rats compared with control animals.

### 4.4. Transforming Growth Factor β (TGF-β)

TGF-β overproduction is the key mediator of fibrosis. Significant relationships exist between the TGF-β expression and the degree of kidney fibrosis [125]. Pereira et al. [126] used immunohistochemistry staining to examine TGF-β in the kidneys of mice infected with *T. gondii*. Increased immunoexpression of the cytokine was observed at 7, 15, 30, and 60 dpi compared with the control group of animals. The authors also noted fibrosis based on increased collagen deposition in the kidneys of mice at 15-, 30-, and 60-days post-*T. gondii* infection. It is suggested that fibrosis is a consequence of increased TGF-β synthesis caused by the presence of *T. gondii*.

### 4.5. Biochemical Parameters in the Serum

The renal panel, including urea and creatine levels, in mice infected with *T. gondii* was performed by Al-Kaysi et al. [127] using the urease-Berthelot method. The authors observed an 8 times higher urea level in the serum of mice experimentally infected with *T. gondii*, whereas creatine level in the infected and uninfected mice was similar. To the contrary, Kudo et al. [120] examined serum blood urea nitrogen (using GLDH methods with an N-assay BUN-L kit) and serum CREA (using a CRE-S kit) in the mice infected with *T. gondii*, and the authors found no significant changes between infected and uninfected hosts. The results obtained by Kudo et al. [120] indicated that the glomerular function and the renal tubular reabsorption ability were maintained in the mice infected with *T. gondii*.

### 4.6. Histopathological Changes

Kudo et al. [120] infected mice with 20 cysts of *T. gondii*. The histological study revealed little evidence of inflammation in glomerular and collecting tubular structures. In the kidneys of rats infected with *T. gondii,* Gharadaghi et al. [124] reported tubular degeneration, tubular dilatation, tubular congestion, glomerular injuries, and even necrosis. Pereira et al. [126] noted mononuclear interstitial nephritis, diffusely distributed and degenerative tubules with coalescent vacuoles, and renal corpuscle changes on days 7, 15, and 30 dpi, which intensified on day 60 dpi in the kidneys of *T. gondii* infected mice. Mattos et al. [128] also observed renal corpuscle lesions and nephritis with perivascular inflammatory infiltrate in mice infected with *T. gondii*.

**Figure 3:**
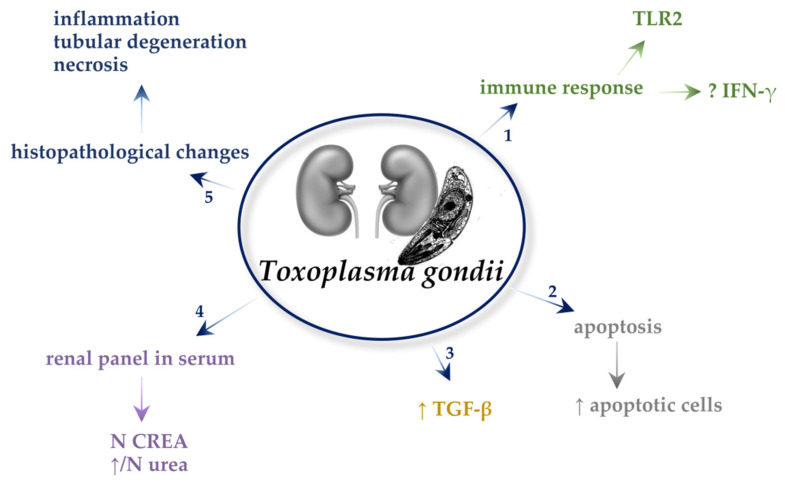
Mechanisms in *Toxoplasma gondii*-infected kidneys. 1. TLR2 has a protective role in kidney function against *T. gondii* infection. While studies about IFN-γ varied, it is reported that IFN-γ inhibits the replication of *T. gondii* in the kidneys and that it does not play a role in the protection of the renal function against *T. gondii* infection. 2. The percentage of apoptotic cells was higher in the kidneys with *T. gondii* infection. 3. *Toxoplasma gondii* cause increased expression of transforming growth factor β (TGF-β). 4. *Toxoplasma gondii* cause no difference in serum creatine (CREA). The urea level may be elevated or in normal ranges. 5. The parasites cause inflammation, tubular degeneration, and necrosis in the kidneys.

All described mechanisms in *T. gondii*-infected kidneys are presented in Figure 3.

## 5. *Acanthamoeba* spp.

*Acanthamoeba* spp. can occur both as free-living organisms in the natural environment, and they can also be facultative pathogens [129].

*Acanthamoeba* spp. can cause cerebral and extracerebral infections of the cornea of the eye, lungs, kidneys, and skin. The infection can develop both in immunocompetent patients and in people with a disturbed immune mechanism [130]. The most common disease caused by free-living amoebas is *Acanthamoeba* keratitis (AK). The infection occurs when swimming in water reservoirs wearing contact lenses and as a result of poor hygiene of contact lenses. *Acanthamoeba* keratitis has also been observed in people with mechanical corneal damage and after refractive procedures [130,131]. In the initial phase of the infection, there is severe eye pain, visual disturbances, photophobia, redness, and foreign body sensation, followed by swelling of the conjunctiva and eyelids [132,133]. A characteristic symptom of the disease is an annular inflammatory infiltrate in the central part of the cornea [133,134]. *Acanthamoeba* spp. also cause granulomatous amoebic encephalitis (GAE). It is an opportunistic, rare disease with a mortality rate of 97–98% [135,136]. Infection occurs by inhalation of air or water aspiration containing invasive forms of *Acanthamoeba* spp. Severe headaches, neck pains, confusion, irritability, hallucinations, fever, nausea, and vomiting have been observed in GAE patients [135,136]. Due to the difficult, often long-term diagnosis of granulomatous encephalitis, treatment of this disease is problematic [129,137].

In the available scientific data base, acanthamoebiasis with infection of amoebas into the kidneys has been found in only one patient of Korean origin who died from meningitis and encephalitis [138]. However, in studies on animals infected with *Acanthamoeba* spp., amoebas were quite often reisolated from kidney samples [139,140]. Knowledge of the mechanisms of *Acanthamoeba* spp. infection into the kidneys is scarce.

### 5.1. Immune Responses

The expression of TLR2 and TLR4 in the kidneys of immunocompetent and immunosuppressed mice infected with *Acanthamoeba* spp. was measured by quantitative real-time polymerase chain reaction (q-real-time PCR) and immunohistochemistry methods by Kot et al. [140]. In the immunocompetent and immunosuppressed uninfected mice, TLR2 expression was found in the proximal tubules, while after *Acanthamoeba* spp. infection brown pigmentation was observed in the distal tubules and collecting ducts. The TLR2 expression was similar in immunocompetent *Acanthamoeba* spp.-infected and uninfected mice. However, in the immunosuppressed mice, there was a statistically significant higher TLR2 expression in mice at 24 dpi compared with the control group and 8 dpi. The statistically significant higher TLR2 levels were noted in immunosuppressed *Acanthamoeba* spp.-infected mice compared with competent *Acanthamoeba* spp.-infected mice [140]. The immunoexpression of TLR4 was observed in the proximal and distal tubules, collecting ducts, and renal corpuscles. The TLR4 expressions between immunocompetent *Acanthamoeba* spp.-infected and uninfected hosts as well as between immunosuppressed infected and uninfected mice were at similar levels. The authors only reported significant differences in the TLR4 expression between the immunocompetent and immunosuppressed mice at 16 and 24 dpi [140]. Based on this study, it is suggested that only TLR2 is involved in response to *Acanthamoeba* spp. infection in the kidneys.

### 5.2. Biochemical Parameters in the Serum and Urine

Steinberg et al. [141] and Brondfield et al. [142] found that hematological and biochemical blood tests in renal transplant patients with disseminated acanthamoebiasis were varied (increased, decreased, or in the normal range). Ringsted et al. [138], in a study on a Korean child with probable GAE and inflammatory lesions in the kidney, found that urine analysis disclosed no abnormality, despite the fact that *Acanthamoeba* spp. were isolated from the kidneys of the patient. Łanocha-Arendarczyk et al. [143] examined biochemical parameters in the serum of immunocompetent and immunosuppressed mice experimentally infected with *Acanthamoeba* spp. The authors did not reveal any significant difference in the levels of CREA, urea, albumin, and total protein in the serum of *Acanthamoeba* spp.-infected immunocompetent and immunosuppressed mice. Łanocha-Arendarczyk et al. [143] also examined Na and K levels as well as CRP level, but the parameters showed no changes between the infected *Acanthamoeba* spp. mice (both immunocompetent and immunosuppressed) and the uninfected mice, and so the results of that biochemical profile were not conclusive in terms of the effect of *Acanthamoeba* spp. on the renal tissue.

### 5.3. Selenium (Se) Concentration

Trace elements, including selenium (Se), modulate the immune function of the host. Se exhibits various effects on the immune system; Se is immunosuppressive in high doses, whereas in low doses, Se is immunostimulating. Selenium is an essential element, which acts as an antioxidant at the cellular level, and it is a cofactor of glutathione peroxidase (GPx), selenoprotein P, and thioredoxin reductase [144]. The imbalance in the metabolism of trace elements can lead to metabolic disturbances and pathophysiological processes [145]. Some researchers suggest that increased and/or decreased concentrations of elements, including Se, might be a potential diagnostic marker indicating, for example, the ongoing neoplastic process in the body [146]. It was found that in parasitic infections, including infections by *Trypanosoma* spp. and *Cryptosporidium* spp., Se levels were elevated and/or decreased in key detoxification organs [147,148]. It is suggested that Se supplementation decreases the parasitemia level of various parasitic infections and reduces parasite-induced organ damage [148].

Selenium concentration in the kidneys of immunocompetent and immunosuppressed mice infected with *Acanthamoeba* spp. was assessed by Łanocha-Arendarczyk et al. [143] using spectrofluorimetry. The concentration of Se in the kidneys of studied mice was as follows: immunosuppressed *Acanthamoeba* spp.-infected mice > immunosuppressed uninfected mice > immunocompetent *Acanthamoeba* spp.-infected mice > immunocompetent uninfected mice. Nephric Se level in the immunocompetent mice was statistically higher in the infected group compared with the control. The authors reported a significantly higher Se concentration in the kidneys of immunosuppressed mice than in immunocompetent infected hosts [143].

### 5.4. Histopathological Changes

Górnik and Kuźna-Grygiel [139] observed various pathomorphological changes in the kidneys of mice infected with different strains of *Acanthamoeba* spp. Bloody ecchymoses and slight inflammation were observed in the kidneys of mice infected with strains isolated from a recreational swimming pool in Szczecin (northwest Poland). In animals infected with a strain isolated from the Goplana Lake in Szczecin, extensive necrotic changes in the tubules and glomeruli were found. In kidney preparations of mice infected with *Acanthamoeba* spp., in whom the amoebae from in vitro cultures had not been reisolated, only small outbreaks were recorded for inflammation in the cortical part of the kidney [139]. Both macroscopic and microscopic changes in the kidneys were observed in dogs with disseminated acanthamoebiasis [149]. The authors observed enlargement of the kidneys with the presence of red ones and yellow nodules located irregularly under the subcapsular surface, and in microscopic slides numerous *Acanthamoeba* spp. trophozoites within the peri-glomerular region were observed. Dubey et al. [150] observed inflammatory infiltrates consisting of macrophages, lymphocytes, and neutrophils and a few amoebas in the kidneys of a dog infected with *Acanthamoeba* spp.

**Figure 4:**
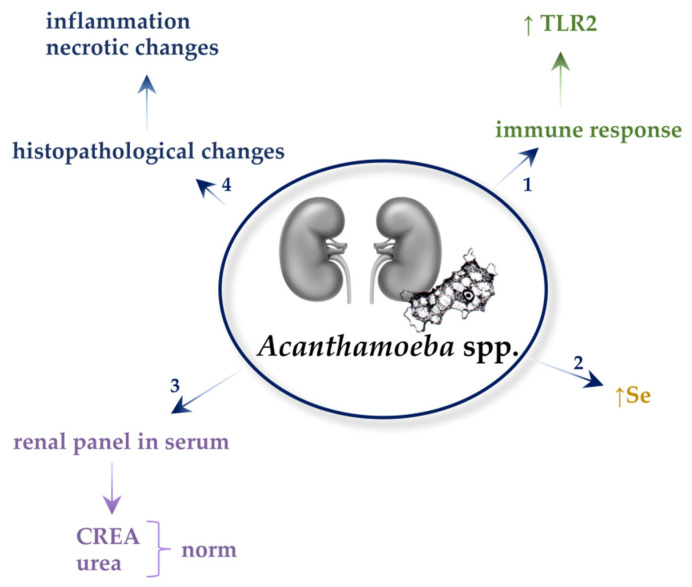
Mechanisms in the *Acanthamoeba* spp.-infected kidneys. 1. *Acanthamoeba* spp. increase the expression of Toll-like receptor 2 (TLR-2). 2. *Acanthamoeba* spp. increase selenium (Se) concentration in the kidneys of infected host. 3. *Acanthamoeba* spp. do not affect the levels of creatine (CREA) and urea in the serum of hosts. 4. The parasites cause inflammation and necrotic changes in the tubules and glomeruli of the kidneys.

All described mechanisms in *Acanthamoeba* spp.-infected kidneys are presented in Figure 4.

## 6. Conclusions and Future Perspectives

The molecular and biochemical mechanisms involved in the response to protozoan parasitic infection in the hosts provide valuable information on the pathogenesis of nephropathy associated with these parasitic infections. The presented mechanisms allow us to understand interactions between the parasites and hosts. Parasites caused inflammation in the kidneys through many processes, including the host’s immune response, oxidative stress, apoptosis, hypoxia, and expression of metalloproteinases. The mechanisms of *Leishmania* spp. and *Plasmodium* spp. infections have been deeply investigated, while *Toxoplasma gondii* and *Acanthamoeba* spp. infections in the kidneys are not well known yet. The presented work may help to find mechanisms that should be examined in kidneys infected with *T. gondii* or *Acanthamoeba* spp. Presenting and understanding the mechanisms in kidneys with *Leishmania* spp. and *Plasmodium* spp. infections may contribute to limiting the degree of tissue damage and to developing an effective pharmacological therapy for treatment of the presented protozoan parasitic infections.
